# Bioaugmentation and vermicompost facilitated the hydrocarbon bioremediation: scaling up from lab to field for petroleum-contaminated soils

**DOI:** 10.1007/s11356-024-32916-8

**Published:** 2024-03-22

**Authors:** Sandra Curiel-Alegre, Aqib Hassan Ali Khan, Carlos Rad, Blanca Velasco-Arroyo, Carlos Rumbo, Rafael Rivilla, David Durán, Miguel Redondo-Nieto, Eduard Borràs, Daniele Molognoni, Soledad Martín-Castellote, Blanca Juez, Rocío Barros

**Affiliations:** 1 International Research Center in Critical Raw Materials for Advanced Industrial Technologies (ICCRAM), University of Burgos, Centro de I+D+I. Plaza Misael Bañuelos S/N. 09001, Burgos, Spain; 2 Research Group in Composting (UBUCOMP), Faculty of Sciences, University of Burgos, Plaza Misael Bañuelos S/N. 09001, Burgos, Spain; 3 Department of Biotechnology and Food Science, Faculty of Sciences, University of Burgos, Plaza Misael Bañuelos S/N. 09001, Burgos, Spain; 4 Department of Biology, Faculty of Sciences, University Autónoma of Madrid, Darwin 2, 28049 Madrid, Spain; 5 Circular Economy & Decarbonization Department, LEITAT Technology Center, Carrer de La Innovació, 2. 08225, Terrassa, Barcelona Spain; 6 ACCIONA, C/ Valportillo II, 8. 28108, Madrid, Alcobendas Spain

**Keywords:** Bioaugmentation, Microbial consortium, Hydrocarbons, Vermicompost, Passive bioelectrochemical systems

## Abstract

The biodegradation of total petroleum hydrocarbon (TPH) in soil is very challenging due to the complex recalcitrant nature of hydrocarbon, hydrophobicity, indigenous microbial adaptation and competition, and harsh environmental conditions. This work further confirmed that limited natural attenuation of petroleum hydrocarbons (TPHs) (15% removal) necessitates efficient bioremediation strategies. Hence, a scaling-up experiment for testing and optimizing the use of biopiles for bioremediation of TPH polluted soils was conducted with three 500-kg pilots of polluted soil, and respective treatments were implemented: including control soil (CT), bioaugmentation and vermicompost treatment (BAVC), and a combined application of BAVC along with bioelectrochemical snorkels (BESBAVC), all maintained at 40% field capacity. This study identified that at pilot scale level, a successful application of BAVC treatment can achieve 90.3% TPH removal after 90 days. BAVC’s effectiveness stemmed from synergistic mechanisms. Introduced microbial consortia were capable of TPH degradation, while vermicompost provided essential nutrients, enhanced aeration, and, potentially, acted as a biosorbent. Hence, it can be concluded that the combined application of BAVC significantly enhances TPH removal compared to natural attenuation. While the combined application of a bioelectrochemical snorkel (BES) with BAVC also showed a significant TPH removal, it did not differ statistically from the individual application of BAVC, under applied conditions. Further research is needed to optimize BES integration with BAVC for broader applicability. This study demonstrates BAVC as a scalable and mechanistically sound approach for TPH bioremediation in soil.

**Supplementary Information:**

The online version contains supplementary material available at 10.1007/s11356-024-32916-8.

## Introduction

Contamination of environmental matrices (including soil and water), both from various anthropogenic and natural processes, poses growing environmental threats (Abdullah et al. 2020; Qurban et al. 2021). Among the anthropogenic sources, oil refineries, chemical manufacturing plants, and gas stations handle and store large quantities of petrochemicals, which can be among the main soil pollution culprits, if improperly managed (Adipah 2019; Truskewycz et al. 2019). In addition, inadequate management, and unregulated dumping of liquid or solid wastes into the open environment, further exacerbates the total petroleum hydrocarbon (TPHs)-associated environmental contamination (Gautam et al. 2023). The biodegradation of TPHs exhibits a general trend from simpler to more complex molecules, with unbranched alkanes and lower molecular weight compounds being more readily degraded (Kebede et al. 2021). However, variations exist within each category depending on specific structures and environmental factors (Imam et al. 2019). The TPH-polluted soils are known to induce human and environmental health emergencies; however, the intensity varies with reference to the routes of exposure (Yousaf et al. 2022). Direct and prolonged exposure to soil TPHs can pose several health risks to humans, including cancer, headache, nausea, fatigue, eye irritation, and skin rash, and can also induce the food chain contamination associated with the grazing livestock, wildlife, and plant-eating insects (Grifoni et al. 2020; Nawaz et al. 2024). Finally, the effect of the loss on soil quality is reflected by a reduction in the presence and activity of different soil microorganisms (Curiel-Alegre et al. 2022a; Truskewycz et al. 2019).

Numerous methods are adopted for the remediation of hydrocarbon in soil. Among these adopted treatments, the use of biological-based remediation systems is considered soft and environmentally friendly (Curiel-Alegre et al. 2022b). Bioremediation offers a promising solution for TPH removal, but translating lab-scale successes to larger scales remains a challenge. (Khan et al. 2016; Yousaf et al. 2022). This is because it is very difficult to maintain the optimal environmental conditions and availability of essential nutrients at field scale that results in limited transfer of these applied biotechnological methods from the levels of lab studies to the full-fledged application stage. Other factors that influence the microbial TPH biodegradation capacity include soil composition, water retention capacity, particle size, and enzyme activities (Martínez-Álvarez et al. 2020). It is not wrong to conclude that many such minor details are pivotal for the application of biotechnological interventions to perform efficient removal and mineralization of organic pollutants. Chen et al. (2019) advocate for a systematic approach to hydrocarbon bioremediation, encompassing thorough assessment, targeted planning, site preparation, bioaugmentation, nutrient supplementation, ongoing monitoring, and final compliance checks. This holistic process aims to efficiently restore contaminated sites and environmental health while adhering to regulatory standards. Hence, once the good results are obtained at a laboratory scale, it must be upscaled in pilot tests. This will enable the stakeholders of the environment (including practitioner, consultants, scientists, and policy makers) to understand and conclude whether, under usual ambient environmental conditions, similar outcomes are achievable, comparable to those that were obtained at lower technology readiness levels (TRL). Further research is necessary to fully comprehend the consequences of this microbial inoculation on the ecosystem during bioremediation since specific organisms introduced to the environment may subsequently form biological pollution via recombination or other mechanisms (Ayilara and Babalola 2023). Studies conducted on a pilot scale have demonstrated that bioremediation can be successful in decreasing TPH pollution and hastening soil recovery (Akbari and Ghoshal 2014; Nobili et al. 2022).

The limitations of individual bioremediation methods can be minimized by co-application with other potential treatment strategies that work on different mechanisms. One of such biological-based remediation approaches is the bioelectrochemical systems (BES). This biotechnology utilizes the electrogenic bacterial processes (biological and electrochemical activities) for the treatment and management of pollution, notably in soil and water. The BES-mediated remediation of TPH and metal-associated pollution in soil can be performed with simultaneously, syntrophic, and cooperative interactions with the microbial populations involved in the bioremediation (Ambaye et al. 2023). The most basic type of electro-bioremediation technique is almost certainly the microbial electrochemical snorkel (MES), which is a non-polarized, electrically conductive object used to short-circuit redox gradients. In soil, MES can provide short-circuiting conditions between the oxic environment outside with the anoxic soil or sediment. This can result in accelerating the rate of biogeochemical reactions by facilitating electron transfer between the microbial population of oxic and anoxic zones, and the possibility of enhancing the degradation of organic matter and pollutants by providing alternative electron pathways (Cruz-Viggi et al. 2022). The primary approach in this recently developed technique to provide the oxygen needed for the biodegradation of hydrocarbons in the soil is oxygen diffusion (Jin and Fallgren 2022). This electron transfer can span centimeters, giving microbes in the soil or sediment remote access to O_2_ that would otherwise be inaccessible (Aulenta et al. 2021; Hoareau et al. 2019).

The combined application of BES along with other biological treatment methods can enhance the remediation of TPH-contaminated soil by providing an electron acceptor to the microbial community (Wang et al. 2021). Existing research on TPH bioremediation highlights the potential of bioelectrochemical systems (BES) as electron acceptors but has not explored their combined application with optimized bioaugmentation and organic amendments at larger scales (Curiel-Alegre et al. 2022b). This study fills this gap by investigating the scaling up of these combined technologies under optimal operative conditions in 500-kg mesocosms. This study addresses this gap by investigating the feasibility of scaling up a combined bioremediation approach in larger mesocosms by co-applying bioelectrochemical systems (BES) with optimized bioaugmentation and organic amendment (vermicompost). The present study aims to identify their impact on both chemical and physical soil properties and biological changes (including soil metagenomics and enzymatic assays) to offer a comprehensive perspective on effective TPH bioremediation options. Further, comprehensive analysis was provided for valuable insights into the effectiveness of TPH bioremediation option for real-world applications.

## Materials and methods

### Soil site properties

Historically, TPH-polluted soil was collected from a machinery park in Noblejas (Toledo, Spain). The sources of contamination were various fuel spills, engine motor oils, and lubricant leaks from parked vehicles and heavy machinery. After excavation, the soil was placed in three separated containers where the respective remediation activities were carried out.

### Chemical reagents

Standards for hydrocarbons (HC) were purchased from LGC Standards Ltd (Teddington, UK): “Alkanes-Mix 12,” 100 µg mL^−1^ in toluene, covering the range C8–C40 (total 14 HCs), and “PAHs-Mix 9” with 19 PAHs according to EPA Method 610 (acenaphthene, acenaphthylene, anthracene, benzo(a)anthracene, benzo(a)pyrene, benzo(b)fluoranthene, benzo(k)fluoranthene, benzo(g,h,i)perylene, chrysene, dibenzo(a,h)anthracene, fluoranthene, fluorene, indeno(1,2,3-c,d)pyrene, naphthalene, phenanthrene, and pyrene) at 100 µg mL^−1^ in acetonitrile. Certified soil reference materials “CRM-357” (sandy loam soil) and “CRM-359” (clay soil) were purchased from Sigma-Aldrich. Chromatographic separation was performed using Isolute Sorbent EPH extraction cartridges (5 mL g^−1^).

### Production of inoculum and nutrient solutions

Microbial consortium was previously isolated from the contaminated soil under study, according to the procedure described by Garrido-Sanz et al. (2019) and Curiel-Alegre et al. (2022b). In 1 L of sterile liquid minimum salt medium (MM), supplemented with 1 mL L^−1^ of phosphate-buffered mineral medium salts (PAS), 0.005% (w/v) yeast extract, and 1 mL L^−1^ of diesel oil (added as a sole carbon and energy source), the microbial consortium was pre-cultured (Hussain et al. 2018; Khan et al. 2017). The consortium was incubated for 5 days at 28 °C, at 200 rpm, before it was scaled up to 20 L, using four separate 25-L containers. These cultures were incubated for 2 weeks at 28 °C, with periodic shaking (Filipič et al. 2015). Once the inocula were grown, representative samples were extracted from each and their optical density and colony-forming units (CFUs mL^−1^) on Plate Count Agar (PCA) were determined.

### Experimental setup

The experiment was carried out at a pilot scale using 500-kg mesocosms for 90 days. Three different experimental conditions were assayed: (a) Control treatment (CT) to assess the natural attenuation of polluted soil, (b) bioaugmentation treatment with vermicompost (BAVC): This involved inoculation and nutrient incorporation into a 2% (w/w) vermicompost-soil mixture, and (c) bioaugmentation treatment with vermicompost and passive MES (BESBAVC): This was identical to BAVC, but with the addition of a passive MES in the form of six 50-cm graphite rods (19 mm diameter) spaced 30 cm apart (GraphiteStore.com, Inc., USA). Nutrients (KH_2_PO_4_ 0.6116; NH_4_NO_3_ 3.0454; and 0.0153 FeCl_3_, in g kg^−1^ of dry soil, respectively) were added as salts to achieve the same concentrations as in Curiel-Alegre et al. (2022b); nitrogen contribution from vermicompost was subtracted from fertilization to avoid overdosing. For all the prepared mesocosm treatments, vermicompost (for BAVC and BESBAVC) and nutrient salts were thoroughly mixed using a shovel. Mesocosms were irrigated with water and microbial suspensions using a phytosanitary backpack sprayer to reach 40% of water retention capacity (WRC) and stored in 1-m^3^ containers in an outdoor shed. Bioaugmentation treatments (BAVC and BESBAVC) received 40 L of microbial culture inoculation each (Supplementary Fig. 1). All treatments, including CT, were irrigated with water to maintain 40% WRC. After 1 month, all mesocosms were re-inoculated with an equal amount of inoculant or water, increasing the water content to reach 50% WRC. During the experiment, the mesocosms were irrigated once or twice a week with water to maintain optimum humidity. For continuous monitoring of soil physical parameters (including temperature and humidity), each treatment was equipped with sensors, GS1 Probes by ProCheck, Decagon, USA, for soil water content monitoring, as done by Şahin et al. (2024), and Tinytag Plus 2, GEMINI Data loggers, UK, for temperature, as done by Vivanco et al. (2024). The results of continuous monitoring were presented in Supplementary Fig. 2.

### Soil sample collection and physicochemical parameters and metal(loid) content analyses

The experiment was conducted for 90 days (Supplementary Fig. 3). Triplicate soil samples were collected using a 7-cm-diameter soil auger (Eijkelkamp, The Netherlands) at T0 (before and after inoculation), T1 (7 days after the inoculation), T2 (30 days after inoculation and second inoculation), T3 (60 days after the inoculation), and T4 (90 days after the inoculation). Fresh soil samples were maintained in refrigerated conditions in the lab after being sieved through a 2-mm mesh. Later, the soil sample was divided into two portions; one is frozen at − 20 °C and the other one was air-dried at ambient temperature, respectively. The frozen and sieved soil samples were used for the quantifying the presence of cultivatable bacteria and extraction of metagenomic DNA (Chaudhary et al. 2021). Air dried soil samples were used for physicochemical analyses, including soil pH and electrical conductivity, determined in a soil water suspension (1:5 w/v), total C and N, measured by dry combustion in a TruSpec (LECO) autoanalyzer (following Marks et al. 2020), organic C, analyzed by wet oxidation using K_2_Cr_2_O_7_ and back-titration with Fe(SO_4_)_2_(NH_4_)_2_ (Mingorance et al. 2007), lime content, quantified by volumetry after acidic attack (Kargas et al. 2022), and soluble N-NO_3_ and exchangeable N-NH_4_ and available PO_4,_ extracted with 1 M KCl and 0.5 M NaHCO_3_, respectively, and analyzed colorimetrically, using a segmented flow analyzer San + (SKALAR, The Netherlands), as done by Curiel-Alegre et al. (2022b). All chemical measurements were made in triplicate. To analyze the changes in available metal(loid)s, Mehlich extraction was performed using Mehlich 3 soil extractant (Al Souki et al. 2021). The extract was then analyzed using ICP-OES (Genesis Spectro, AMETEK, Germany). Precision and accuracy of the analyses were assured and maintained by including a multi-standard of 21 elements of known concentration (ICP multi-element standard solution—89,166.180, VWR, Germany) and blanks (1 for every 15 samples). The raw average results are presented in supplementary information (Supplementary Table 1). The raw data was used for network analysis, which is detailed in the “Statistical analysis” section.

### Bacterial consortium biodiversity, sequencing, and analysis

Bacterial biodiversity was studied by the analysis of the 16S RNA gene. For this purpose, DNA was isolated in triplicate from soil samples. Total DNA was extracted from one gram of homogenized sample soil using the FastDNA Spin Kit for Soil (MP Biomedicals, USA) according to manufacturer indications. The isolated DNA was quantified using Qubit 4 fluorometer (Invitrogen). Genomic DNA 16S rDNA region was amplified by PCR using the primers 27F (5′-AGAGTTTGATCMTGGCTCAG-3′) and 1492R (5′-CGGTTACCTTGTTACGACTT-3’), as suggested by Sagar et al. (2014). Each sample was amplified in triplicate and multiplexed by using a set of 27F and 1492R primers with a specific barcode tail for each sample. After barcoding PCR, the amplicons were pooled, and 100 femtomoles were utilized for library preparation using Oxford Nanopore Ligation Sequencing Kit (SQK-LSK114) and NEBNext® Companion Module for Oxford Nanopore Technologies® Ligation Sequencing (cat #E7180S) according to their specifications. A total of 12 femtomoles of the multiplexed libraries were loaded and subsequently sequenced on Flongle Flow Cel (R10.4.1, ONT) using the MinION Mk1B device. Data was acquired, base-called, and quality-filtered using MinKNOW version 22.03.4 (ONT).

### EPH quantification

EPHs were extracted, following the method described by Curiel-Alegre et al. (2022b). Soil extraction was performed from 1 g of dried soil sample and 20 mL of a mixture acetone-hexane (1:1, v/v) in a microwave oven (Ethos X, Milestone, Italy) at 150 °C for 20 min. After centrifugation (30 min at 2500 *g*), the supernatant was filtered (0.22 µm) and evaporated to a volume of 1 mL on a rotary evaporator (SAVANT SPD111V, Thermo). Extracted EPHs were fractionated using Isolute EPH cartridges (25 mL/5 g) previously conditioned with 30 mL hexane, at ambient pressure, and eluted with 12 and 20 mL of hexane and DCM (dichloromethane) at a flow rate of 2 to 3 mL min^−1^, for the elution of aliphatic and aromatic hydrocarbons, respectively. The fractions obtained were evaporated using a stream of N_2_ to above 1 mL and injected on a Varian 3900 gas chromatograph (GC) equipped with a flame ionization detector (FID) device and a Varian CP8907 capillary column (25 m, 0.25 mm inner diameter, with a film thickness of 0.25 mm). The splitless injection was carried out with a temperature of 250 °C and a volume of 3 µL. The initial temperature of the oven was 80 °C, rising to 200 °C at 7 °C min^−1^, then reaching 300 °C at 11 °C min^−1^, which was maintained for 17 min. Helium was the carrier gas (74 kPa). The FID operated at 325 °C and 20 Hz. The hydrocarbon decontamination process was evaluated by determining the degradation yield for each soil sample, through the following equation (Micle and Sur 2021):

$$\upeta =\frac{{C}_{{\text{i}}}-{C}_{{\text{f}}}}{{C}_{{\text{i}}}}100\; [\%]$$

where η is the yield, in percentage; *C*_f_ is EPHs concentration in the soil at the end of the treatment time, in mg kg^−1^; and *C*_i_ is initial EPHs concentration in the soil, in mg kg^−1^.

### Soil enzyme activities

The studied activities were analyzed with fluorogenic 4-methylumbelliferone (MUF) or 7-amino-4-methylcoumarin (AMC) substrates in 96-microtiter plates using a fluorometric plate reader (GENios, TECAN) with 360/450 nm excitation/emission filters, respectively (Marx et al. 2001). These measured hydrolytic activities were acid phosphatases (EC 3.1.3.2—AcPA), alkaline phosphatase (EC 3.1.3.3—AlkPA), α-glucosidases (EC 3.2.1.20—aGA), β-glucosidases (EC 3.2.1.21—bGA), *N*-acetyl-β-glucosaminidase (EC 3.2.1.30—bNAG), β-xylosidase (EC 3.2.2.27—bXyl), leucine-aminopeptidase (EC 3.4.11.1—LeuAMP), and sulfatase (EC 3.1.6.1—AS).

### Statistical analysis

For statistically analysis of data, three separate experimental samples were used. Normality and homogeneity of variances were then assessed using Kolmogorov’s and Levene’s tests, respectively. The data were analyzed with a unidirectional ANOVA (meaning a significant difference at *p* < 0.05 for treatment versus control for each time). Significant differences between means were obtained using Tuckey’s test. Statistical analysis was performed using Prism 8.0 (GraphPad). Demultiplexing and high-quality base-calling of the soil metagenomic data were carried out using SUP algorithm of Guppy 6.0.7. Operational taxonomic units (OTUs) were obtained by clustering raw reads at an 80% similarity threshold using VSEARCH 2.22.1 (Rognes et al. 2016). OTU taxonomic assignment was conducted using the DADA2 assign Taxonomy function (Callahan et al. 2016) and the SILVA SSU 138 database (Quast et al. 2013). Network analysis was conducted using the Extended Bayesian Information Criterion Graphical Least Absolute Shrinkage and Selection Operator (EBICglasso) method. The Fruchterman-Reingold algorithm visualized the network, positioning nodes (metals and metalloids), and edges (interrelationships) according to their connection strength (Mushtaq et al. 2020; Saleem et al. 2023). The network analysis was performed on JASP (v 0.18.1.0). Statistical analysis of genomic data, including diversity metrics and community structure assessments, was performed using R packages Phyloseq, (McMurdie and Holmes 2013), Microbiome (v 0.99.41), and Vegan: Community Ecology Package (v 2.6–4).

## Results

### Characterization of properties of soil and organic amendment

Polluted soil of the machinery park has been excavated from different areas with oil spills, mixed, divided in three mesocosms, and analyzed. A commercial vermicompost (Vermicultura Duque S.L., Madrid, Spain) was chosen as organic amendment for the BAVC and BESBAVC treatments, at a rate of 2% (w/w). The physicochemical properties of the vermicompost used are as follows: organic matter 40% (w/w), pH 7.2, organic N 1.5%, soluble P_2_O_5_ 1.5%, exchangeable K_2_O 1%, Mg 1%, Fe 1.5%, Mn 356 mg kg^−1^, Cu 100 mg kg^−1^, total humic acids 20%, and bacterial load 10^10^ g kg^−1^. The analytical properties of the three mesocosms are summarized in Table 1. Statistically significant difference among the different mesocosms was noted in case of humidity, organic matter, soluble N-NO_3_, and initial EPHs, compared to the control treatment.

**Table 1:** Basic properties and hydrocarbon concentration of soil mesocosms at the beginning of the experience

Basic properties	CT	BAVC	BESBAVC
Humidity (%)	0.36 ± 0.02 b	4.28 ± 0.81 b	10.74 ± 2.64 a
Field capacity (%)	28.22 ± 0.01 a	24.80 ± 0.26 a	24.80 ± 0.19 a
Total carbon (%)	6.21 ± 0.09 a	5.75 ± 0.11 a	5.98 ± 0.13 a
Total nitrogen (%)	0.04 ± 0.00 a	0.03 ± 0.00 a	0.03 ± 0.01 a
Electrical conductivity (dS m^−−1^)	0.49 ± 0.08 a	0.41 ± 0.12 a	0.39 ± 0.09 a
pH (1:5 H_2_O)	8.35 ± 0.04 a	8.61 ± 0.03 a	8.35 ± 0.01 a
Organic matter (%)	10.38 ± 2.00 a	8.51 ± 1.75 ab	7.34 ± 1.46 b
CaCO_3_ (%)	68.13 ± 8.75 a	75.27 ± 11.47 a	69.31 ± 9.28 a
Soluble N-NO_3_ (mg kg^−1^)	1.32 ± 0.05 b	3.88 ± 0.90 a	2.72 ± 0.72 a
Extractable N-NH_4_ (mg kg^−1^)	18.64 ± 0.74 a	22.75 ± 1.36 a	17.45 ± 1.25 a
Available P-PO_4_ (mg kg^−1^)	1.75 ± 0.07 a	0.56 ± 0.15 b	0.80 ± 0.10 a
EPHs (mg kg^−1^)	40,533 ± 1665 a	34,113 ± 519 b	30,793 ± 645 b
PAHs (mg kg^−1^)	0.18 ± 0.01 a	0.26 ± 0.07 a	0.16 ± 0.00 a

### Evolution of soil properties

**Fig. 1:**
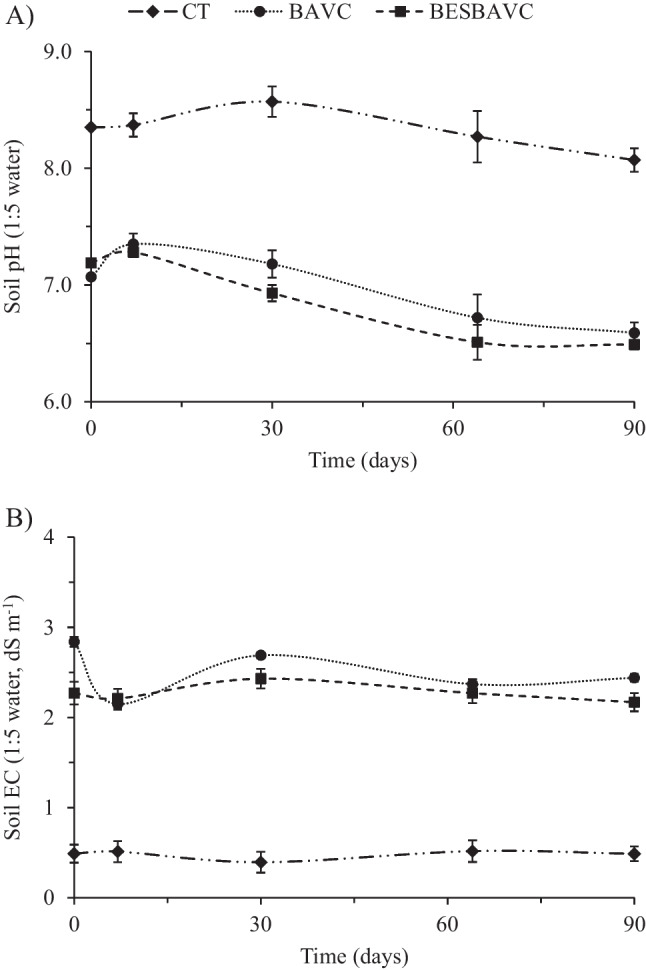
Soil physiochemical parameters. **A** pH and **B** electrical conductivity during the pilot scale experience

**Fig. 2:**
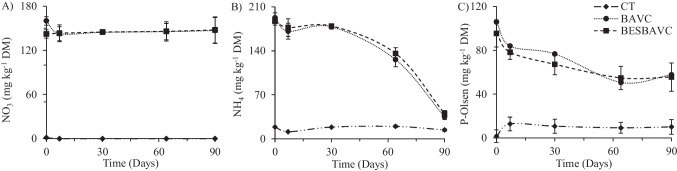
Nutrients evolution (mg kg^−1^ DW) at the pilot scale experiment: **A** nitrates, **B** ammonium, and **C** orthophosphate

During the 90 days of the study, humidity and temperature data were recorded, which are shown in Supplementary Fig. 2. Temperature and atmospheric humidity have been monitored with sensors placed in the air environment and under the ground at 20 cm depth, and results were similar, with minimum temperatures of 15.8 °C and a maximum of 40.4 °C for the CT and BAVC treatments, which in the case of the BESBAVC treatment reached 50.8 °C; it could be due to graphite rods incorporated in this treatment, as graphite is a good conductor of heat and electricity. Regarding the humidity data for the CT, BAVC, and BESBAVC treatments, the minimum recorded were 30.5% RH (relative humidity), 0% RH, and 12.1% RH, respectively, with a maximum of 100% RH. The soil properties’ evolution was studied through the results obtained from the soils at different sampling times. There were no significant differences in soil pH values (Fig. 1A) in the CT throughout the experiment. In the treatments with bioaugmentation (BAVC and BESBAVC), a small rise in pH is shown at the beginning of the experiment, followed by an acidification that will be related to the microbial respiration due to the addition of the microbial consortia, vermicompost, or nutrients. The electrical conductivity results (Fig. 1B) of CT and BESBAVC soil treatment showed a sightly variation during the 90 days of the experiment. BAVC treatment had significant variations in the first days before stabilizing on day 30. It was observed that the CT treatment had a much lower electrical conductivity than the two bioaugmentation treatments (BAVC and BESBAVC) due to the absence of organic amendment. Both bioaugmentation treatments have shown similar values in the evolution of available orthophosphate, ammonium, and nitrate levels compared to the CT treatment, as shown in Fig. 2. Figure 2B shows the most significant difference; a large decrease in ammonium values is observed, probably due to ammonium consumption by the microbial community. The rest of the physicochemical analyses showed no significant differences to be considered in future real scale-ups.

### Evolution of biological parameters

**Fig. 3:**
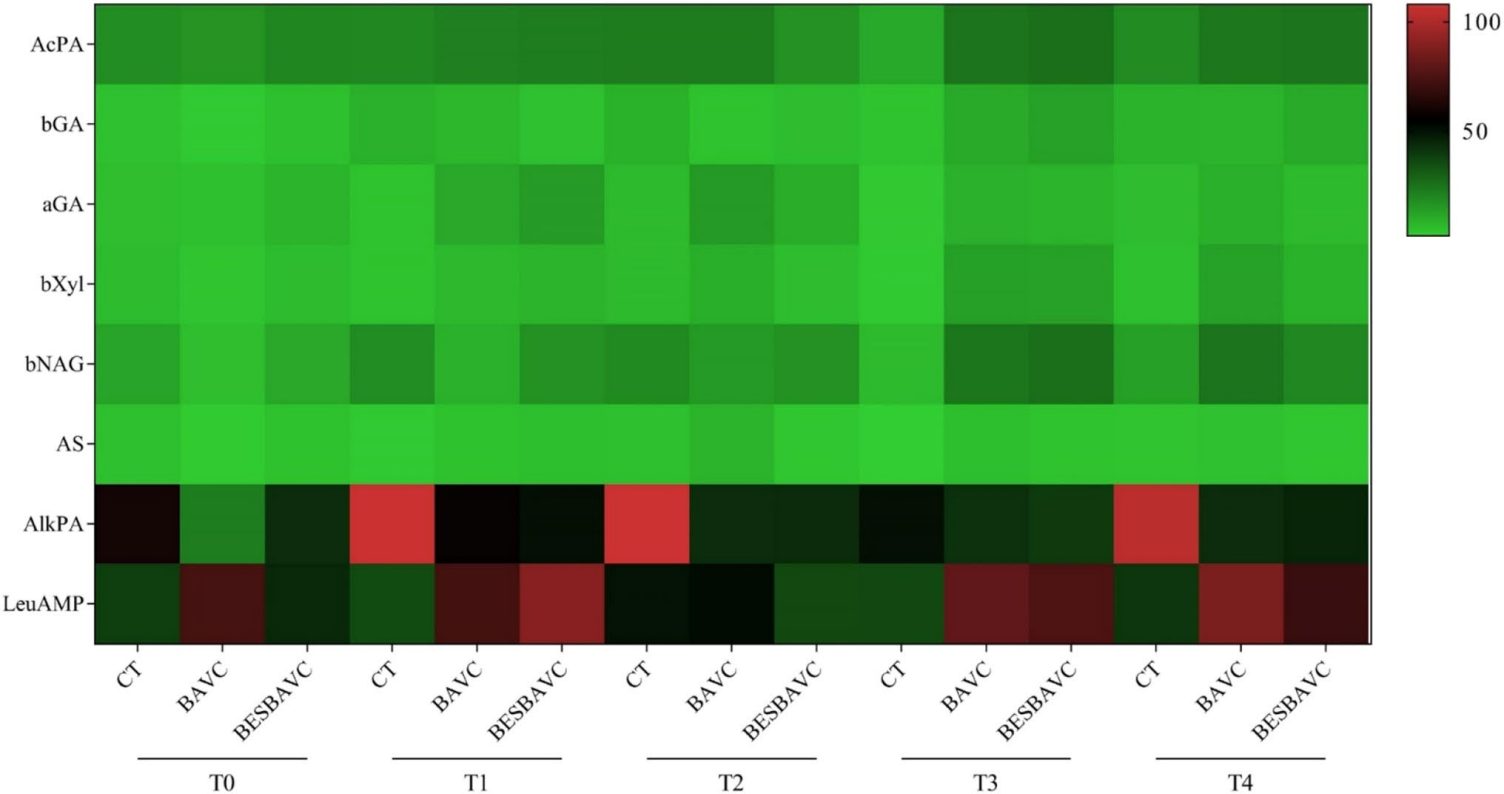
Heat map of the enzyme activities of the three treatments (CT, BAVC, and BESBAVC) throughout the different sampling points (T0, 0 days; T1, 7 days; T2, 30 days; T3, 60 days; and T4, 90 days). Green indicates low values and red indicates high values

Complementary to the physicochemical analysis, hydrolytic enzyme activities were evaluated to understand the soil microbial activity evolution, as shown in Fig. 3. For further information, the enzyme activity profile of the samples was analyzed for five intervals (T0, T1, T2, T3, and T4); the values are provided in Supplementary Table 2. After 7 days of incubation (T1), an increase in microbial activity began, which continued until day 60 (T3), where it stabilized and stopped moderately, with a further increase at 90 days (T4). For the TC treatment, a significant growth of phosphatases (alkPA) was observed, which is not common in treatments without the addition of microbial consortia, because this enzyme activity is related to bacterial growth. In the case of treatment with bioaugmentation (BAVC and BESBAVC), however, microbial growth is observed in all genera that form the synthetic community, with proteases (LeuAMP) being predominant for both treatments. These results showed an increase in the catabolic capacities of the microbial community.

### EPHs and PAHs biodegradation

**Fig. 4:**
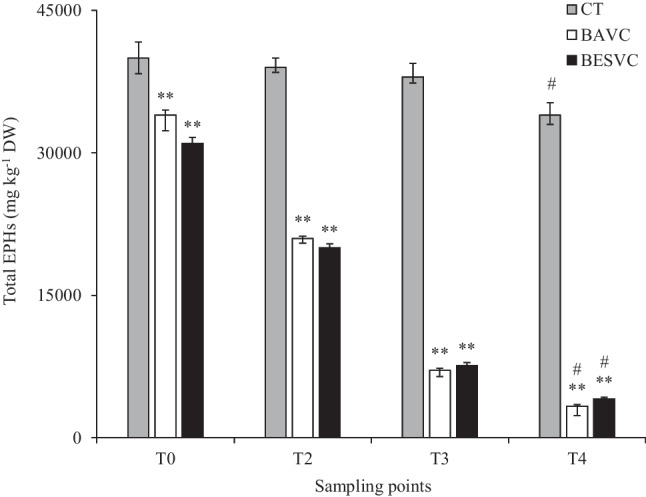
Content of EPHs during the pilot scale experience. Columns with different symbols showed significant statistical differences, asterisks (**) meaning significant difference at *p* < 0.05 for treatment versus control for each time, while number sign (#) showing significant differences at *p* < 0.05 for time zero versus other sampling time points (T0, 0 days; T2, 30 days; T3, 60 days; and T4, 90 days)

EPHs were analyzed from the initial time to 90 days of experience, also including analysis for days 30 (T2) and 60 (T3). These analyses were carried out to know the hydrocarbon degradation efficiency of the tested treatments. Evaluation of the effectiveness of the bioremediation treatments, through the degradation percentages for EPHs, was obtained at the end of the experience, after 90 days (T4). In addition, the variation of the EPHs throughout the experience can be observed in Fig. 4, and details are provided in Supplementary Table 3. For CT treatment, the EPHs content (mg kg^−1^) at 90 days (T4) was 34,000, which was initially at 40,000, while for BAVC, it is reduced to 3300 mg kg^−1^, from initial levels of 34,000, and in the case BESBAVC treatment, it is reduced from 31,000 to 4100 mg kg^−1^, which represent a degradation yield (%) of 15.0, 90.3, and 86.8 for each treatment, respectively, compared with the initial content. The three study strategies have shown a decrease in EPHs levels over time; for the bioaugmentation treatments (BAVC and BESBAVC), significant differences have been obtained with respect to the control (CT) at each sampling point. The BESBAVC treatment showed a more progressive and continuous decrease in EPH degradation during the experiment, compared with the BAVC treatment, whose decrease was less abrupt and stabilized over time. In addition, significant differences were observed at 90 days of the experiment, if the 0-day value is compared with the other sampling points. The 16 EPA (Environmental Protection Agency) PAHs for each sampling period, from the start of the experiment to the end at 90 days, including 30 and 60 days, were analyzed and are shown in Table 2. All the treatments studied showed a decreasing trend, and at the end of the experiment, the values were undetectable. Phenanthrene with a concentration of 32 µg kg^−1^, fluoranthrene with 32 µg kg^−1^, and pyrene with 120 µg kg^−1^ were found in the highest amounts at time 0, also becoming undetectable at the last sampling point.

**Table 2:** Concentration of 16 EPA PAHs from the initial contaminated soil (T0) to the different sampling points (T2, T3, and T4) expressed in mg kg^−1^

Treatment ID	CT	BAVC	BESBAVC	CT	BAVC	BESBAVC	CT	BAVC	BESBAVC	CT	BAVC	BESBAVC
Sampling point	T0			T2			T3			T4		
Naphthalene	< 0.010	< 0.010	< 0.010	< 0.010	*	< 0.010	*	*	< 0.010	*	*	*
Acenaphthylene	< 0.010	< 0.010	< 0.010	< 0.010	*	0.014	*	*	< 0.010	*	*	*
Acenaphthene	< 0.010	< 0.010	< 0.010	< 0.010	*	< 0.010	*	*	< 0.010	*	*	*
Fluorene	< 0.010	< 0.010	< 0.010	< 0.010	*	< 0.010	*	*	< 0.010	*	*	*
Phenanthrene	0.032	< 0.010	< 0.010	< 0.010	*	0.013	*	*	< 0.010	*	*	*
Anthracene	< 0.010	< 0.010	< 0.010	< 0.010	*	< 0.010	*	*	< 0.010	*	*	*
Fluoranthene	0.036	< 0.010	< 0.010	< 0.010	*	0.030	*	*	< 0.010	*	*	*
Pyrene	0.120	0.078	0.076	< 0.010	*	0.051	*	*	0.014	*	*	*
Benzo(a)anthracene	< 0.010	< 0.010	< 0.010	< 0.010	*	< 0.010	*	*	< 0.010	*	*	*
Chrysene	< 0.010	< 0.010	< 0.010	< 0.010	*	< 0.010	*	*	< 0.010	*	*	*
Benzo(b)fluoranthene	< 0.010	< 0.010	< 0.010	< 0.010	*	< 0.010	*	*	< 0.010	*	*	*
Benzo(k)fluoranthene	< 0.010	< 0.010	< 0.010	< 0.010	*	< 0.010	*	*	< 0.010	*	*	*
Benzo(a)pyrene	< 0.010	0.180	< 0.010	< 0.010	*	< 0.010	*	*	< 0.010	*	*	*
Dibenzo(a,h)anthracene	< 0.010	< 0.010	< 0.010	< 0.010	*	< 0.010	*	*	< 0.010	*	*	*
Benzo(g,h,i)perylene	< 0.010	< 0.010	< 0.010	< 0.010	*	< 0.010	*	*	< 0.010	*	*	*
Indeno(1,2,3-c,d)pyrene	< 0.010	< 0.010	< 0.010	< 0.010	*	< 0.010	*	*	< 0.010	*	*	*
HAP 16 EPA	0.180	0.260	< 0.160	< 0.160	*	< 0.160	*	*	< 0.160	*	*	*

*Non-detectable fraction

### Metal interaction network

**Fig. 5:**
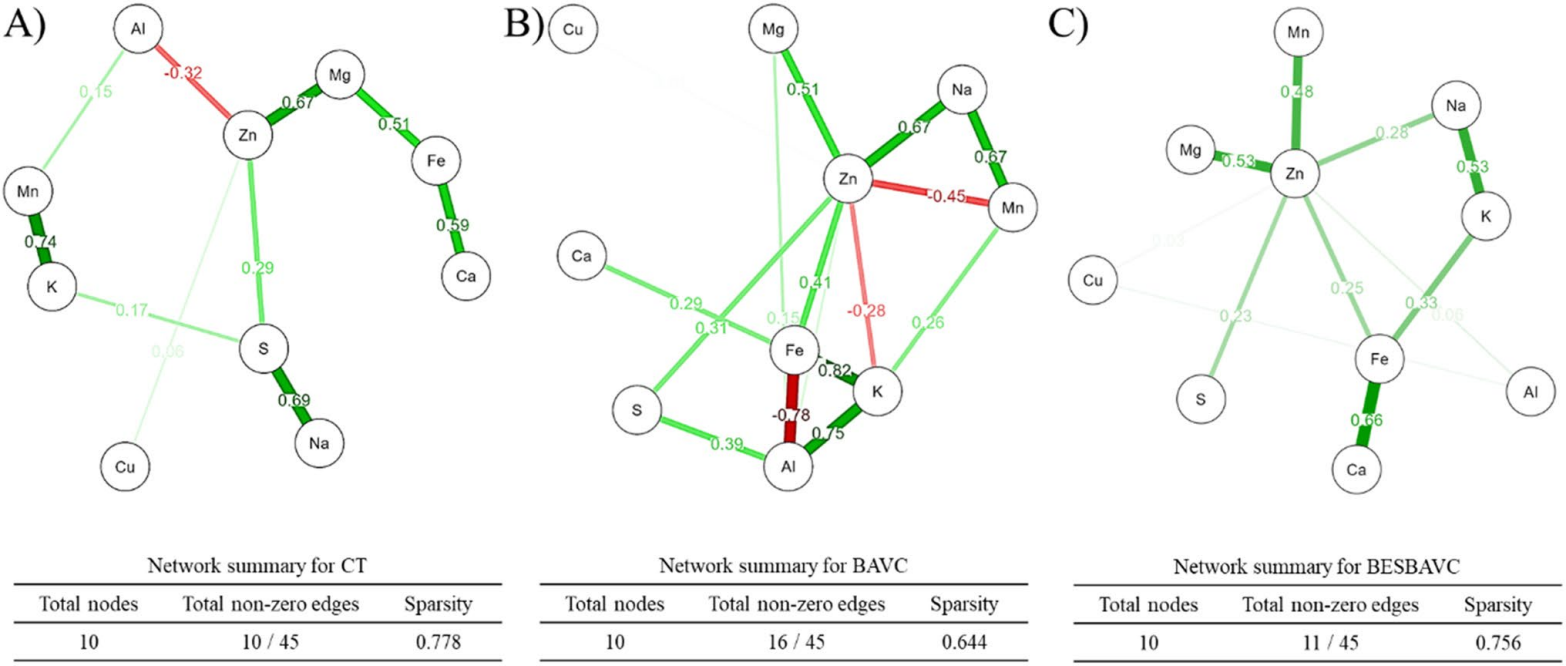
Network analysis performed using EBICglasso estimator based on Fruchterman-Reingold algorithm to assess the correlation pattern in reference to different applied treatments. **A** Control, **B** bioaugmentation and vermicompost (BAVC), and **C** bioaugmentation, vermicompost, and passive snorkel bioelectrochemical system (BESBAVC)

The results of Mehlich-3 extractable metal(loid)s are shown in Supplementary Table 1. Using raw data, a network analysis was done to understand relations between available metal and metalloids (Fig. 5), while the details of the weight matrix used for the preparation of these networks are presented in Supplementary Table 4 and Supplementary Table 5. Specifically, the study found that the maximum number of possible edges between the metals was 45. However, the network analysis showed that only a subset of these edges was present in each of the treatment groups. The CT treatment group had 10 edges, the BAVC treatment group had 16 edges, and the BESBAVC treatment group had 11 edges. The sparsity of the network, which is a measure of how spread out the edges are, was also found to be different for each treatment group. The CT treatment group had the highest sparsity (0.778), followed by the BESBAVC treatment group (0.756) and the BAVC treatment group (0.644). It indicates that only 22 to 35% of interaction between the metals was observed. Zn in all treatment was showing the highest number of edges/interactions, with 4 in CT (positive interaction with Mg, S, and Cu, while negative with Al), 8 in BAVC (positive interaction with Fe, Mg, Na, S, and Cu, while negative with K and Mn), and 7 in BESBAVC (all positive with Al, Fe, Mg, Mn, Na, S, and Cu). While most interactions were positive, few negative interactions were also noted, including between Zn and Al (− 0.32) in CT, Zn and K (− 0.45), Zn and Mn (− 0.45), and Fe with Al (− 0.78). In the case of BESBAVC, no negative interaction was noted, suggesting that in this treatment no antagonist behavior was observed in the metal with increment in time. Based on the analysis, it can also be concluded that there were no significant differences between CT, BAVC, and BESBAVC, based on the sparsity (around 0.6 to 0.7 in all cases).

### Evolution of the bacterial community during bioremediation

Before inoculation, the inoculant and the vermicompost used were evaluated for bacterial density by determining CFU mL^−1^ or CFU g^−1^. The inoculant that showed an OD_600_ of 1.2, contained over 10^8^ CFU mL^−1^ while the vermicompost showed ca. 10^4^ CFU g^−1^, indicating that the contribution of vermicompost to the bacterial community was negligible. Soil samples were also assessed for bacterial abundance and the values were typically between 10^7^ and 10^8^ CFU g^−1^, indicating a suitable bacterial community in the soil for biodegradation. Furthermore, the inoculated mesocosms (BAVC and BESBAVC) had a similar, although slightly higher, number of CFU g^−1^ than the uninoculated control treatment.

**Fig. 6:**
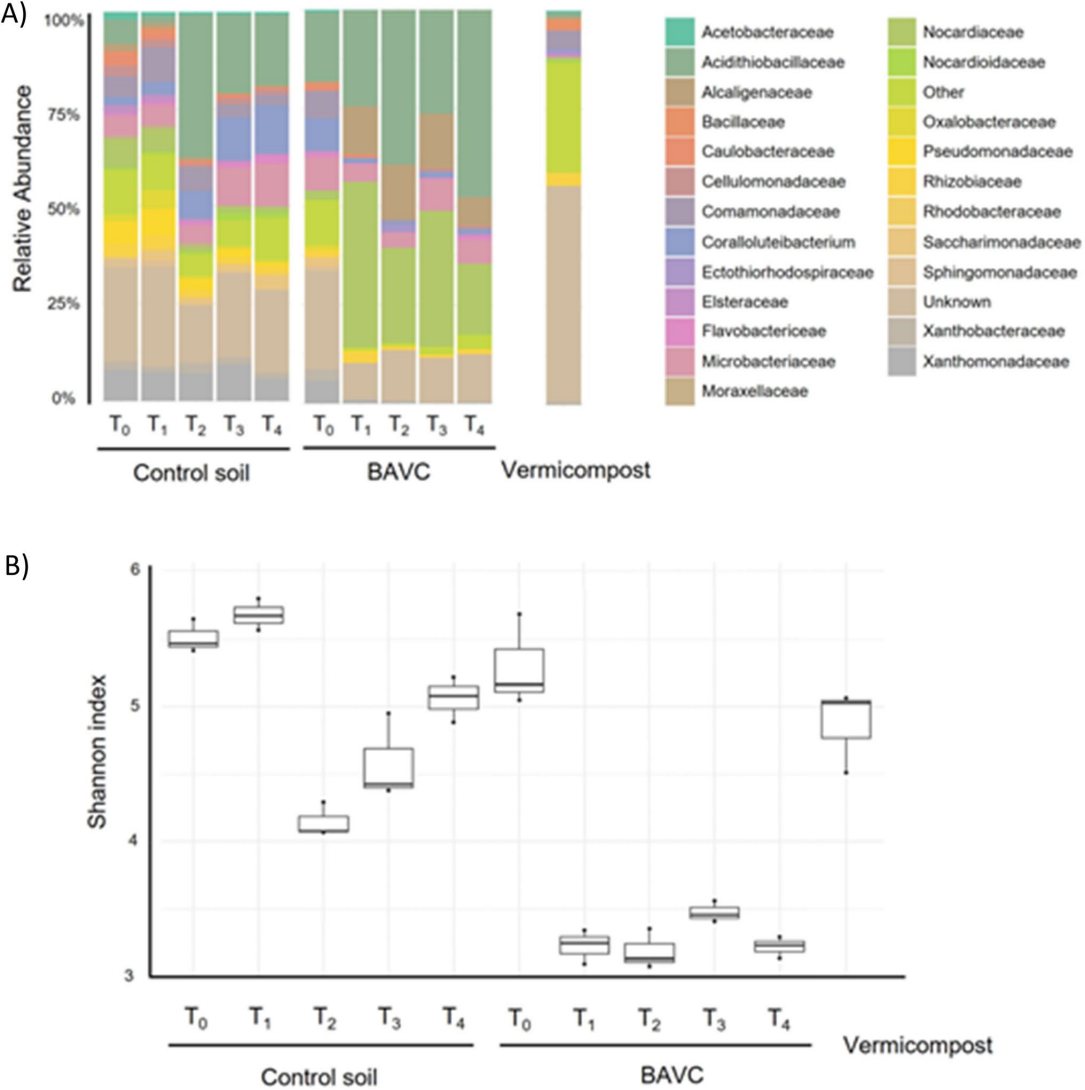
Compositional analysis of the control and treated soil communities, **A** bacterial relative abundances at the level of family for the different samples at the different sampling times. Relative bacterial OTUs abundance at the level of family of the mesocosms at each of the timepoints for the 23 most abundant families. The remaining classes were grouped under the category Other. The barplots represent the sum of OTUs from the three replicates. **B** Boxplot representing the bacterial Shannon index values for each timepoint and sample. Differences between Shannon index values at the different timepoints were assessed with the Kruskal–Wallis test. Three independent biological replicates of each sample were analyzed

**Fig. 7:**
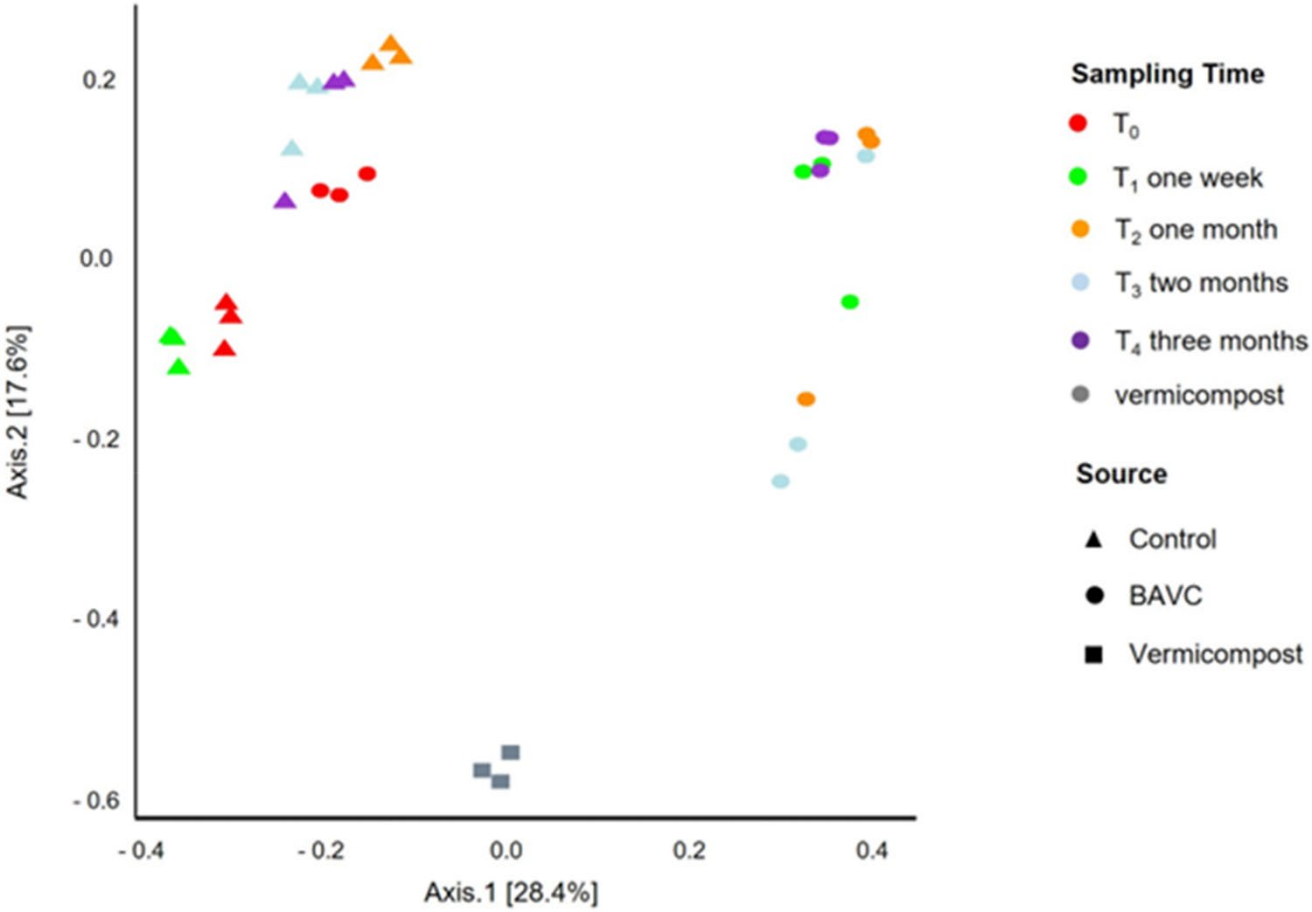
Clustering analysis of the bacterial communities within CT and BAVC at the different timepoints. Principal coordinate analysis (PCoA) of mesocosms using Bray–Curtis distances. Colors according to timepoint, and shapes according to mesocosm. Averages for each mesocosms and sampling time are represented

As there were no statistically significant variations in biodegradation observed between BAVC and BESBAVC treatments, the evolution of the microbial community throughout the bioremediation process was only studied and compared between the CT and BAVC treatments. This evolution study is constructed using the amplicon sequencing of 16S rRNA, in three replicates for the control and BAVC samples, in the five timepoints (T0 to T4), as shown in Fig. 6A. The taxonomy distribution according to the relative bacterial OTUs abundance of the 23 major bacterial families identified indicated a clear variation between the families present in the control and the BAVC sample (Fig. 6A) at times T1 to T4. At T4, more than 50% of the OTUs identified in the BAVC treatment belonged to families *Alcaligenaceae* and *Nocardiaceae*. These families were less predominant in CT sample. It is also evident that the bacterial composition of the vermicompost bacterial population is completely different than that of the soil and due to the limited number of bacteria, it is not expected to have a major influence in the microbial processes in biopiles. As shown in Fig. 6B, alpha-diversity, presented as Shannon index revealed than the control and the BAVC soil, possessed a similar value at T0. However, after the inoculation, Shannon index dropped in all BAVC samples, indicating that the addition of the consortium produced a big impact of inoculation. Furthermore, analysis of beta-diversity (Fig. 7) showed that the communities of the control treatment clustered together with the T_0_ sample of the treated soil, indicating that at the experiment beginning both soils harbored similar bacterial populations, and that this CT community did not have significant changes during the treatment. Conversely, the community in the BAVC-treated contaminated soil strongly changed after inoculation, and this change was maintained through the bioremediation process. The community in the vermicompost was very different from the other two communities.

## Discussion

Bioaugmentation has been widely recognized as an effective procedure used for decontamination of TPH polluted soils (Khan et al. 2017). The use of bioaugmentation technologies using microorganism previously isolated from the TPH polluted soil is an effective way for soil bioremediation. Bakina et al. (2021) demonstrated the effectiveness of the bioaugmentation to enhance hydrocarbon remediation in the oil-contaminated soils. Similarly, Nwankwegu et al. (2022) provided an overview of the advantages of bioaugmentation technologies for soil remediation, as they can be cost-effective treatments, performed in situ, without the need of applying hazardous chemical inputs, and able to achieve high biodegradation levels of organic pollutants if favorable environmental conditions are maintained. However, one of the most notable issues for the application of any bioremediation methods is that when the treatment study is performed at lab scale or at lower TRLs, the result demonstrates significant potential; however, as soon as these findings are translated to higher TRLs, i.e., at field and commercial scale, the efficacy of remediation methods is drastically dropped (Li et al. 2019). Numerous factors influence the rate of remediation. For instance, Martínez-Rivera and Cardona-Gallo (2021), studying the degradation of oil spills in tropical soils, demonstrate the successfulness of bioaugmentation and biostimulation methods combining inoculation with compost, nutrients, surfactants, and other organic amendments, such as leonardite, as promising strategies for the treatment of oil spills. The results presented here showed that soil humectation has a positive impact on bacterial diversity after 7 days of incubation (T2), but this diversity decreased at T3 when humectation was incremented at 50% WRC, possibly by a decrease in aeration. In most cases, despite an initial promise, various studies have highlighted the critical challenge of full-scale bioremediation: the potential for isolated bacteria to experience diminishing enzymatic activity over time, likely due to environmental pressures (Demichelis et al. 2017; Poorsoleiman et al. 2020). Therefore, if a bioremediation treatment demonstrates a thriving soil microbial population and improved enzymatic activities, it becomes a strong candidate for upscaled commercial application. Furthermore, the results of the present study demonstrate the BAVC application potential at upscaled level, as this treatment had significantly higher TPH (aliphatic and aromatic) biodegradation potential and relatively stable soil enzyme activities over time were observed (Table 2, Figs. 3 and 4). The results of the present study also highlight the importance of selecting an adequate bioremediation strategy, depending on the type of hydrocarbon contamination present in the soil.

In the present study, the soil was contaminated not only with diesel oil, also with other motor oils and lubricants, containing higher concentration of long-chain (C21–C35) petroleum hydrocarbon compared to other types petroleum hydrocarbons (Table 4S). Zhang et al. (2019a) studied the feasibility of a biodegradation of hydrocarbon in the contaminated soils of a decommissioned refinery and demonstrated the effectiveness of combined strategies like bioaugmentation-assisted landfarming and BES for the TPH treatment. Their study concluded that the co-applying these bioremediation strategies was the most beneficial approach. However, our current investigation found no statistically significant difference between the two bioaugmentation treatments (BAVC and BESBAVC). Therefore, considering the similar results and potential cost increase, utilizing BES technology may not be advisable in this specific case. The addition of compost has been recognized to be an advantageous approach for the recovery and remediation of contaminated soils, affected with a wide number of pollutants, including hydrocarbon, metal(loid)s, dyes, and pharmaceutical waste (Hussain et al. 2018; Khan and Barros 2023). These organic pollutants include TPHs, aromatic compounds (including the notorious contaminants like BTEX and PAHs), and halogenated hydrocarbons, including chlorophenols, explosives, and pesticides (Khan et al. 2018; Hussain et al. 2022). An organic amendment for soil such as compost can stabilize the soil structure and promote the activity of degrading microorganisms by improving oxygen diffusion, water, and nutrient availability. It is also very inexpensive because it is derived from biodegradable organic wastes; therefore, it can help to the environmental sustainability of remediation procedures (Iqbal et al. 2020; Mustafa et al. 2021).

The use of organic amendments (like compost) positively affects the activity, size, and composition of the soil microbial community, although, their effects were mainly due to the physicochemical characteristics of compost matrix rather than to compost-borne microorganisms (Saison et al. 2006; Velasco-Arroyo et al. 2024). The inoculation with the consortium had a profound impact on the bacterial community of polluted soil, resulting in changes both in alpha and beta diversity; alpha diversity decreased drastically in the inoculated soil because of the predominance of *Nocardiaceae* and *Alcaligenaceae* families that were maintained during the 90 days of incubation (Fig. 6). These results indicate that after inoculation, large changes in bacterial populations arose. Like the present finding, Martínez-Rivera and Cardona-Gallo (2021) also found that biostimulation treatments decreased alpha diversity of the polluted soil due to the increase of Actinobacteria phylum in treated soils. Our results also agreed with the effect of bioaugmentation in Ecopiles shown by Martínez-Cuesta et al. (2023). However, in their case, the predominance between the different bacterial families changed throughout a year of monitoring in response to the environmental changes. The metagenome analyses of the bacterial community, in the present work, suggested that the bacterial population naturally present in vermicompost are not expected to have a major effect on biodegradation, since the bacterial levels are very low in comparison with those already present in soil or with the bioaugmentation consortium and the main role of vermicompost was to provide nutrients and stimulate microbial activities. In turn the soil microbial activities are also linked with the soil enzyme activities; hence, a stable and thriving microbial population is a good indicator of soil health (Khan et al. 2023a, 2023b). In a previous experiment at a lab scale (Curiel-Alegre et al. 2022b), the introduction of 2% (w/w) of vermicompost stimulated hydrocarbon removal in this TPHs’ contaminated soil with a clear increase in basal soil respiration, bacterial biomass, and enzyme activities; in this work, the same results were obtained at a pilot scale, increasing TPH degradation and stimulating other soil biochemical processes such as nitrification and AlkPA and LeuAMP. Koolivand et al. (2020) confirmed the effectiveness of enriching soil and vermicompost mixtures with native bacterial consortia that was isolated from oily petroleum sludge, increased the degradation rates from 31–49% in vermicompost to 85–91% for the bioaugmented vermicompost.

The bioelectrochemical systems (BES) usage is also reported to have significant implications for the field of bioremediation of TPHs’ contaminated soils and other environmental matrices. Studies such as Lan et al. (2023) show BES-based systems as an efficient and biologically responsive method towards the bioremediation of polluted soil. Similarly, they proposed that BES energized and enhanced the anaerobic oxidation of various organic wastes to minimize soil and groundwater contaminants such as petroleum hydrocarbons and halogenated chemicals. While passive BES demonstrated potential for microbial activity in the present study, it fell short of significantly boosting TPH biodegradation. This could be due to several factors, including limited nutrients, a mismatch in the fostered microbial community, or insufficient electron donors (Zhang et al. 2019b). Another reason for limited MES activity could be due to the soil moisture content, as Lu et al. (2014) reported up to 78.7% higher biodegradation with tubular BES in raw water-saturated soils containing petroleum hydrocarbon. Therefore, further investigation into microbial composition, nutrient availability, and BES optimization could shed light on these limitations and pave the way for more effective bioremediation strategies.

The aim of this research was to establish the most appropriate techniques for upscaling the bioremediation treatment. Based on the prior research, microbial consortia immobilized on plant-based residues increase hydrocarbon breakdown efficiency after 30 days, demonstrating relevance for beneficial impact owing to growth-promoting transporter and extra supply of C and N (Tao et al. 2019). However, the presented results were primarily associated with soil respiration and the changes due to enzymatic activity (Pacwa-Płociniczak et al. 2019; Meyer et al. 2018). In the present study, for the first 30 days, the crude oil (TPHs) undergoes degradation by microbial activity. The activation of alkane-degrading enzymes is likely to be linked to an increase of nutrients, particularly amino compounds (Curiel-Alegre et al. 2022b). Similar finding was reported by Haleyur et al. (2019) who observed that with longer incubation time, the removal of TPHs and PAHs, along with the role of biostimulation, gains importance, while the influence of bioaugmentation becomes less significant to the overall TPH biodegradation process. This might be in concordance with the quantitative reduction of nutrients and the increase of intermediary metabolites in the edaphic growth medium which can affect ecotoxicity, and translating into reduced viability in eukaryotic organisms as shown in the results. One way to enhance the degradation of hydrocarbon in soil is to add biosurfactants. While *Pseudomonas* sp. can enhance TPH decontamination of the soil through rhamnolipid production under bioremediation treatments (Ramadass et al. 2018), further investigation is needed to determine its specific role and potential limitations (including commercial and economic) in the context of BAVC treatment upscaling and co-application.

This study employed a customized microbial consortium alongside organic amendments (specifically vermicompost for nutrient supplementation and microbial activation) to validate the effectiveness of bioaugmentation in enhancing the remediation of soil contaminated with TPHs. Furthermore, this work contributes to the growing body of research exploring the potential application of bioelectrochemical systems in soil bioremediation. While BES demonstrate promise, the present study concluded that further assessments are required to ensure cost-effectiveness across diverse scenarios. A unique feature of this study is its focus on evaluating the feasibility of scaling up TPH bioremediation for real-world applications. The generated information offers valuable insights for environmental practitioners and policymakers seeking to develop sustainable and efficient strategies for remediating TPH-contaminated soils. Ultimately, these findings contribute to advancements in knowledge within the field of soil bioremediation, potentially guiding future research endeavors and paving the way for improved remediation methodologies applicable to various environmental contaminants, including the legacy (accumulated toxic metal(loid)s, poly-halogenated aromatic hydrocarbons, and persistent organic pollutants) and emerging pollutant (including, but not limited to, pharmaceuticals and personal care products, nanomaterials, and endocrine-disrupting chemicals).

## Conclusions

The optimized bioaugmentation treatments, both with the application of an organic amendment (BAVC) and with a microbial electrochemical system (BESBAVC), showed promising results after 90 days in comparison to the natural attenuation having no added nutrient, suggesting their potential as green, sustainable, efficient, and potentially cost-effective solutions for soil bioremediation. Both BAVC and BESBAVC significantly improved soil EPH degradation compared to the control treatment. However, no significant difference was observed between BAVC and BESBAVC, suggesting that BAVC might be a more cost-effective option. Therefore, it is expected that the BAVC technique will be used to bring this experience to full scale, since it has achieved very promising results and with lower economic costs than BESBAVC. After 90 days, higher concentrations of long-chain hydrocarbons (C21-C30 and C30-C35) persisted in the treated soil, as expected with the presence of motor oil and lubricant contamination. These fractions are more challenging to degrade compared to hydrocarbons in gasoline or diesel oil. Optimal temperature, humidity, and aeration were crucial for achieving significant TPH biodegradation by the isolated microbial community in the bioaugmentation treatments. The soil, naturally contaminated with TPHs from motor oils and other types of hydrocarbons, harbored a metabolically active microbial population capable of hydrocarbons degradation. The bioaugmentation TPHs degrading microbial community along with vermicompost addition and, in some case, BES implementation can further enhance TPH biodegradation rates.

## Supplementary Information

Below is the link to the electronic supplementary material.

Supplementary file1 (DOCX 1896 kb)

## Data Availability

Authors have mentioned in the Consent for publication that Data and materials would be available on request.
