# Supplementary material for: Bioaugmentation and vermicompost facilitated the hydrocarbon bioremediation: scaling up from lab to field for petroleum-contaminated soils
Source: Environ Sci Pollut Res Int. 2024 Mar 22;32(28):16601–16. doi: 10.1007/s11356-024-32916-8 (PMC12325483; doi:10.1007/s11356-024-32916-8)
Supplement: Supplementary file 1 — Supplementary file1 (DOCX 1896 kb) [file 11356_2024_32916_MOESM1_ESM.docx]

**Supplementary information**

Hydrocarbon Bioremediation: Scaling Up from Lab to Field for Petroleum-Contaminated Soils

Sandra Curiel-Alegre^a,b^, Aqib Hassan Ali Khan^a^, Carlos Rad^b^, Blanca Velasco-Arroyo^c^, Carlos Rumbo^a^, Rafael Rivilla^d^, David Durán^d^, Miguel Redondo-Nieto^d^, Eduard Borràs^e^, Daniele Molognoni^e^, Soledad Martín-Castellote^f^, Blanca Juez^f^, Rocío Barros^a*^

^a^ International Research Center in Critical Raw Materials for Advanced Industrial Technologies (ICCRAM). University of Burgos, Centro de I+D+I. Plaza Misael Bañuelos s/n. 09001 Burgos, Spain.

^b^ Research Group in Composting (UBUCOMP). University of Burgos, Faculty of Sciences. Plaza Misael Bañuelos s/n. 09001 Burgos Spain.

^c^ Department of Biotechnology and Food Science, University of Burgos, Faculty of Sciences. Plaza Misael Bañuelos s/n. 09001 Burgos Spain.

^d^ Department of Biology, Faculty of Sciences, University Autónoma of Madrid, Darwin 2, 28049 Madrid, Spain

^e^ LEITAT Technology Center, Circular Economy & Decarbonization Department, Carrer de la Innovació, 2. 08225, Terrassa, Barcelona, Spain.

^f^ ACCIONA, C/ Valportillo II, 8. 28108, Alcobendas, Madrid, Spain.

***Corresponding authors:** [rbarros@ubu.es](mailto:rbarros@ubu.es)

**
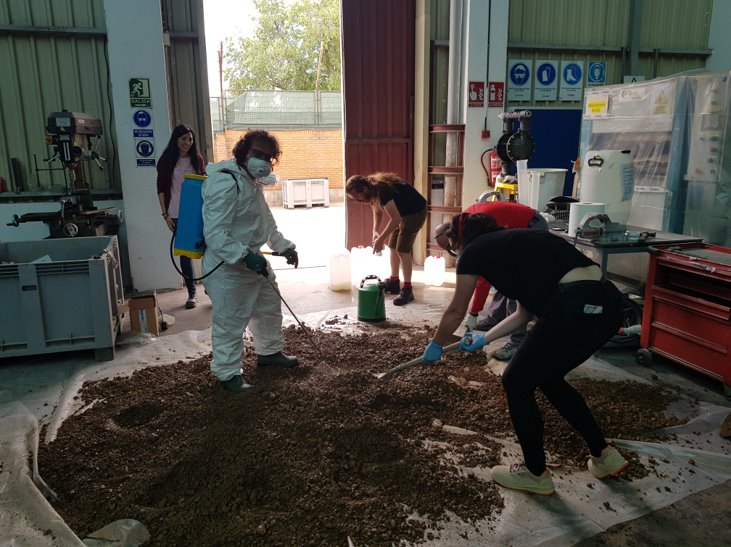
**

**Supplementary Figure 1.** The application of nutrients and bacterial suspension at the start of experiment during first addition of bacterial inoculants spray.


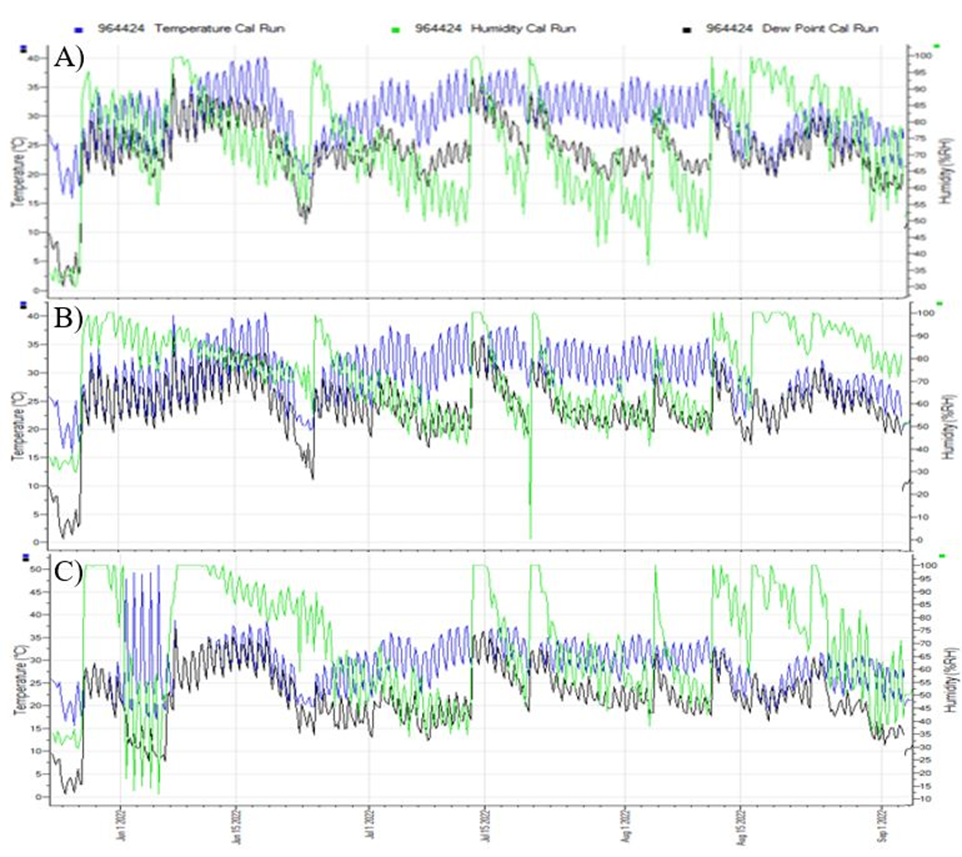


**Supplementary Figure 2.** Results of monitorization for temperature and moisture of: A) CT treatment, B) BAVC treatment, and C) BESBAVC treatment.


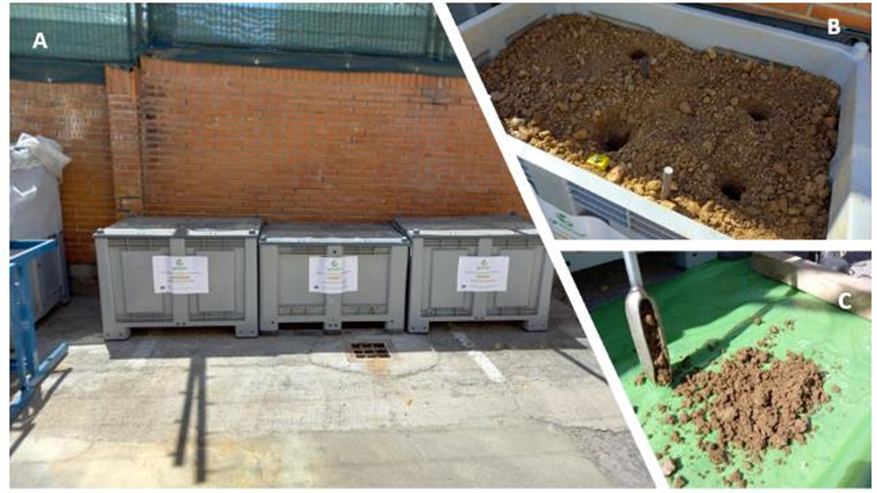


**Supplementary Figure 3.** Pilot scale in ACCIONA´s Facilities - off site (Alcobendas, Madrid): ​A) three containers for treatments under study, B) Visualization of soils under study, and C) sample collected using auger.

**Supplementary Table 1.** The evolution of extractable metals in the soil of applied treatment with reference to time (0, 7, 30, 60, and 90 days)

| **CT** | **Al** | **Ca** | **Fe** | **K** | **Mg** | **Mn** | **Na** | **S** | **Cu** | **Zn** |
| --- | --- | --- | --- | --- | --- | --- | --- | --- | --- | --- |
| **T0** | 0.299 ± 0.110 | 3182 ± 12 | 12.44 ± 0.57 | 10.06 ± 0.75 | 110.98 ± 0.14 | 7.58 ± 0.23 | 98.49 ± 5.80 | 11.87 ± 0.78 | 0.232 ± 0.041 | 5.211 ± 0.031 |
| **T7** | 0.267 ± 0.005 | 2577 ± 62 | 11.47 ± 0.43 | 7.53 ± 0.27 | 102.19 ± 2.17 | 11.53 ± 0.31 | 106.86 ± 5.41 | 17.11 ± 0.56 | 0.308 ± 0.021 | 5.242 ± 0.190 |
| **T30** | 1.359 ± 0.015 | 2862 ± 151 | 14.29 ± 1.02 | 16.82 ± 0.34 | 109.75 ± 10.48 | 21.50 ± 3.15 | 105.28 ± 12.24 | 16.03 ± 1.54 | 0.291 ± 0.082 | 4.228 ± 0.301 |
| **T60** | 0.236 ± 0.064 | 2373 ± 289 | 11.17 ± 0.84 | 8.1 ± 0.62 | 98.88 ± 0.11 | 12.81 ± 1.70 | 103.50 ± 7.43 | 14.50 ± 1.93 | 0.315 ± 0.020 | 4.401 ± 0.219 |
| **T90** | 2.837 ± 0.096 | 2997 ± 128 | 9.06 ± 0.90 | 13.92 ± 0.90 | 104.19± 9.59 | 17.95 ± 6.24 | 104.01 ± 8.67 | 19.82 ± 2.37 | 0.340 ± 0.015 | 4.349 ± 0.211 |
| **BAVC** | **Al** | **Ca** | **Fe** | **K** | **Mg** | **Mn** | **Na** | **S** | **Cu** | **Zn** |
| **T0** | 0.130 ± 0.025 | 3647 ± 73 | 12.17 ± 0.94 | 40.19 ± 2.89 | 111.58 ± 0.68 | 9.85 ± 0.24 | 95.79 ± 2.12 | 118.19 ± 6.10 | 0.180 ± 0.005 | 3.578 ± 0.086 |
| **T7** | 0.158 ± 0.017 | 3478 ± 36 | 12.54 ± 0.38 | 33.67 ± 0.49 | 109.76 ± 1.02 | 12.19 ± 0.20 | 101.63 ± 10.42 | 122.39 ± 3.87 | 0.197 ± 0.021 | 3.549 ± 0.061 |
| **T30** | 2.515 ± 0.017 | 3674 ± 128 | 9.42 ± 1.75 | 14.21 ± 1.44 | 112.64 ± 5.73 | 11.54 ± 2.57 | 90.91 ± 5.44 | 123.28 ± 7.15 | 0.294 ±0.016 | 3.601 ± 0.472 |
| **T60** | 0.239 ± 0.068 | 3247 ± 129 | 10.14 ± 0.51 | 37.65 ± 2.26 | 97.40 ± 3.15 | 12.46 ± 0.65 | 94.16 ± 8.92 | 129.74 ± 12.32 | 0.214 ± 0.018 | 3.456 ± 0.192 |
| **T90** | 0.701 ± 0.214 | 3100 ±191 | 7.28 ± 0.85 | 12.80 ± 0.96 | 105.19 ± 4.86 | 10.66 ± 1.50 | 100.46 ± 8.85 | 103.91 ± 4.88 | 0.345 ± 0.007 | 3.190 ± 0.954 |
| **BESBAVC** | **Al** | **Ca** | **Fe** | **K** | **Mg** | **Mn** | **Na** | **S** | **Cu** | **Zn** |
| **T0** | 0.229 ± 0.052 | 3404 ± 77 | 13.06 ± 0.28 | 43.35 ± 0.33 | 109.46 ± 0.04 | 11.99 ± 0.17 | 110.13 ± 8.25 | 78.80 ± 2.23 | 0.248 ± 0.044 | 4.705 ± 0.096 |
| **T7** | 0.310 ± 0.080 | 3238 ± 57 | 13.57 ± 0.82 | 38.74 ± 0.05 | 109.08 ± 1.65 | 14.36 ± 0.10 | 95.43 ± 4.19 | 108.58 ± 1.57 | 0.236 ± 0.008 | 4.842 ± 0.093 |
| **T30** | 0.406 ± 0.084 | 3236± 32 | 12.73 ± 0.30 | 40.99 ± 1.55 | 100.80 ± 0.29 | 15.14 ± 0.34 | 99.17 ± 14.79 | 105.45 ± 0.69 | 0.242 ± 0.033 | 4.703 ± 0.088 |
| **T60** | 0.176 ± 0.021 | 2929± 29 | 13.721± 0.38 | 35.90 ± 0.38 | 101.67 ± 1.44 | 14.07 ± 0.36 | 99.18 ± 4.63 | 97.36 ± 6.97 | 0.221 ± 0.011 | 4.481 ± 0.093 |
| **T90** | 0.515 ± 0.080 | 3237± 15 | 12.41 ± 0.54 | 34.12 ± 1.13 | 99.34 ± 3.59 | 15.76 ± 0.54 | 102.57 ± 7.17 | 117.19 ± 4.88 | 0.273 ± 0.016 | 5.021 ± 0.120 |

**Supplementary Table 2.** Enzymatic evolution from the initial contaminated soil (T0) to the different sampling points (T0, T1, T2, T3, and T4), expressed in nmol g^-1^ min^-1^.

| Treatment | Soil enzyme | **T0** | | | **T1** | | | **T2** | | | **T3** | | | **T4** | | |
| --- | --- | --- | --- | --- | --- | --- | --- | --- | --- | --- | --- | --- | --- | --- | --- | --- |
| **CT** | **AcPA** | 18.55 | ± | 1.80 | 19.92 | ± | 1.88 | 23.07 | ± | 1.89 | 11.26 | ± | 1.21 | 18.96 | ± | 0.88 |
|  | **bGA** | 5.31 | ± | 0.96 | 9.32 | ± | 1.31 | 9.15 | ± | 0.95 | 4.39 | ± | 0.34 | 8.89 | ± | 1.64 |
|  | **aGA** | 5.98 | ± | 0.25 | 4.47 | ± | 0.28 | 7.11 | ± | 0.51 | 3.27 | ± | 0.19 | 6.16 | ± | 0.47 |
|  | **bXyl** | 6.48 | ± | 0.52 | 4.45 | ± | 0.30 | 7.01 | ± | 0.52 | 3.06 | ± | 0.21 | 5.43 | ± | 0.26 |
|  | **bNAG** | 12.59 | ± | 0.57 | 18.52 | ± | 1.17 | 19.35 | ± | 0.71 | 6.83 | ± | 0.64 | 13.60 | ± | 1.65 |
|  | **AS** | 5.42 | ± | 0.18 | 3.16 | ± | 0.25 | 5.54 | ± | 0.35 | 2.23 | ± | 0.14 | 4.09 | ± | 0.16 |
|  | **AlkPA** | 59.94 | ± | 1.90 | 107.00 | ± | 1.76 | 107.94 | ± | 3.09 | 50.76 | ± | 2.53 | 102.92 | ± | 2.51 |
|  | **LeuAMP** | 39.28 | ± | 2.22 | 35.72 | ± | 1.22 | 50.29 | ± | 2.27 | 36.37 | ± | 2.90 | 40.95 | ± | 1.90 |
| **BAVC** | **AcPA** | 16.99 | ± | 1.07 | 21.97 | ± | 1.91 | 22.97 | ± | 0.62 | 24.92 | ± | 1.81 | 24.38 | ± | 1.76 |
|  | **bGA** | 3.05 | ± | 0.74 | 7.71 | ± | 1.50 | 4.65 | ± | 0.44 | 10.95 | ± | 0.57 | 8.52 | ± | 0.43 |
|  | **aGA** | 5.40 | ± | 0.23 | 11.65 | ± | 0.54 | 15.30 | ± | 0.46 | 9.38 | ± | 0.46 | 9.64 | ± | 0.39 |
|  | **bXyl** | 4.33 | ± | 0.17 | 7.25 | ± | 0.21 | 9.74 | ± | 0.41 | 13.62 | ± | 0.62 | 13.41 | ± | 0.77 |
|  | **bNAG** | 5.97 | ± | 0.59 | 9.24 | ± | 1.47 | 15.30 | ± | 0.93 | 24.62 | ± | 1.88 | 24.82 | ± | 1.31 |
|  | **AS** | 2.84 | ± | 0.26 | 4.86 | ± | 0.23 | 8.19 | ± | 0.29 | 5.65 | ± | 0.29 | 5.09 | ± | 0.15 |
|  | **AlkPA** | 22.60 | ± | 3.45 | 56.81 | ± | 1.76 | 43.27 | ± | 1.79 | 42.61 | ± | 1.54 | 43.35 | ± | 2.86 |
|  | **LeuAMP** | 72.65 | ± | 3.46 | 72.58 | ± | 2.00 | 52.11 | ± | 1.75 | 79.91 | ± | 1.99 | 86.50 | ± | 2.00 |
| **BESBAVC** | **AcPA** | 20.40 | ± | 1.58 | 22.73 | ± | 1.02 | 17.51 | ± | 3.44 | 26.44 | ± | 1.59 | 24.97 | ± | 1.19 |
|  | **bGA** | 5.51 | ± | 0.43 | 5.08 | ± | 0.45 | 6.32 | ± | 0.95 | 13.41 | ± | 1.65 | 11.04 | ± | 0.53 |
|  | **aGA** | 8.49 | ± | 0.28 | 14.91 | ± | 0.61 | 10.15 | ± | 0.63 | 8.40 | ± | 0.48 | 6.62 | ± | 0.21 |
|  | **bXyl** | 6.50 | ± | 0.30 | 8.36 | ± | 0.39 | 6.24 | ± | 0.90 | 13.06 | ± | 0.46 | 9.10 | ± | 0.45 |
|  | **bNAG** | 11.53 | ± | 0.89 | 17.67 | ± | 0.87 | 17.46 | ± | 1.41 | 26.39 | ± | 1.60 | 20.05 | ± | 1.02 |
|  | **AS** | 4.98 | ± | 0.38 | 5.68 | ± | 0.14 | 3.86 | ± | 0.51 | 4.45 | ± | 0.21 | 4.02 | ± | 0.09 |
|  | **AlkPA** | 43.36 | ± | 2.97 | 50.76 | ± | 1.66 | 43.66 | ± | 2.90 | 39.91 | ± | 3.00 | 45.77 | ± | 2.36 |
|  | **LeuAMP** | 44.68 | ± | 1.50 | 89.66 | ± | 2.45 | 36.12 | ± | 2.29 | 74.76 | ± | 2.84 | 69.73 | ± | 2.91 |

**Supplementary Table 3.** Concentration of TPHs from the initial contaminated soil (T0) to the different sampling points (T2, T3, and T4) expressed in mg kg^-1^.

| **Treatment ID** | **Sampling Point** | **EPH C10-C12** | **EPH C12-C16** | **EPH C16-C21** | **EPH C21-C30** | **EPH C30-C35** | **EPH C35-C40** | **Total EPH** |
| --- | --- | --- | --- | --- | --- | --- | --- | --- |
| CT | T0 | <30 | <50 | 430 | 22000 | 15000 | 2000 | 40000 |
| BAVC |  | <30 | <50 | 410 | 19000 | 13000 | 200 | 34000 |
| BESBAVC |  | <30 | <50 | 390 | 17000 | 12000 | 190 | 31000 |
| CT | T2 | <30 | 54 | 410 | 21000 | 16000 | 2100 | 39000 |
| BAVC |  | <30 | <50 | 280 | 12000 | 7700 | 1200 | 21000 |
| BESBAVC |  | <30 | <50 | 300 | 11000 | 7300 | 1100 | 20000 |
| CT | T3 | <30 | <50 | 410 | 21000 | 14000 | 2000 | 38000 |
| BAVC |  | 9 | 52 | 230 | 3300 | 2700 | 530 | 7100 |
| BESBAVC |  | 8 | 45 | 210 | 3900 | 2900 | 430 | 7600 |
| CT | T4 | <30 | <50 | 380 | 19000 | 13000 | 1900 | 34000 |
| BAVC |  | 4,7 | 30 | 120 | 1700 | 1200 | 190 | 3300 |
| BESBAVC |  | 5,6 | 36 | 140 | 2100 | 1500 | 250 | 4100 |

**Supplementary Table 4.** Centrality measures per variable (extractable metals) for different treatments.

| **For Control** | | | | | | | | | |
| --- | --- | --- | --- | --- | --- | --- | --- | --- | --- |
| Variable | | Betweenness | | Closeness | | Strength | | Expected influence | |
| Al |  | -0.204 |  | 0.677 |  | -0.113 |  | -1.315 |  |
| Ca |  | -0.842 |  | -0.443 |  | -0.952 |  | -0.575 |  |
| Fe |  | 0.179 |  | 0.123 |  | 0.446 |  | 0.684 |  |
| K |  | -0.587 |  | -0.681 |  | 0.109 |  | 0.412 |  |
| Mg |  | 0.945 |  | 0.803 |  | 0.824 |  | 1.078 |  |
| Mn |  | -0.715 |  | -0.795 |  | -0.084 |  | 0.233 |  |
| Na |  | -0.842 |  | 0.197 |  | -0.509 |  | -0.163 |  |
| S |  | 0.817 |  | 0.901 |  | 1.342 |  | 1.56 |  |
| Cu |  | -0.842 |  | -2.039 |  | -2.041 |  | -1.588 |  |
| Zn |  | 2.093 |  | 1.258 |  | 0.979 |  | -0.324 |  |
| **For BAVC** | | | | | | | | | |
| Variable | | Betweenness | | Closeness | | Strength | | Expected influence | |
| Al |  | 0.297 |  | 0.581 |  | 0.853 |  | -0.328 |  |
| Ca |  | -0.752 |  | -0.719 |  | -1.194 |  | -0.916 |  |
| Fe |  | -0.227 |  | 0.726 |  | 0.699 |  | -0.59 |  |
| K |  | 0.822 |  | 0.619 |  | 1.107 |  | 1.968 |  |
| Mg |  | -0.752 |  | -0.142 |  | -0.79 |  | -0.227 |  |
| Mn |  | -0.752 |  | 0.113 |  | 4.784×10^-6^ |  | -0.54 |  |
| Na |  | -0.752 |  | 0.176 |  | -0.039 |  | 1.055 |  |
| S |  | 0.647 |  | 0.327 |  | -0.695 |  | -0.064 |  |
| Cu |  | -0.752 |  | -2.521 |  | -1.388 |  | -1.253 |  |
| Zn |  | 2.222 |  | 0.841 |  | 1.448 |  | 0.896 |  |
| **For BESBAVC** | | | | | | | | | |
| Variable | | Betweenness | | Closeness | | Strength | | Expected influence | |
| Al |  | -0.588 |  | -1.105 |  | -0.915 |  | -1.142 |  |
| Ca |  | -0.588 |  | 0.052 |  | -0.193 |  | -0.124 |  |
| Fe |  | 0.334 |  | 0.502 |  | 1.184 |  | 1.116 |  |
| K |  | -0.357 |  | 0.166 |  | 0.224 |  | 0.211 |  |
| Mg |  | -0.588 |  | 0.502 |  | -0.204 |  | -0.281 |  |
| Mn |  | 0.219 |  | 0.605 |  | -0.353 |  | -0.285 |  |
| Na |  | 0.104 |  | 0.454 |  | 0.092 |  | 0.154 |  |
| S |  | -0.588 |  | -0.192 |  | -0.643 |  | -0.563 |  |
| Cu |  | -0.588 |  | -2.246 |  | -1.292 |  | -1.197 |  |
| Zn |  | 2.641 |  | 1.262 |  | 2.101 |  | 2.111 |  |

**Supplementary Table 5.** Weight matric used for the preparation of network plots for variables (extractable metals) for different treatments.

| **For Control** | | | | | | | | | | | |
| --- | --- | --- | --- | --- | --- | --- | --- | --- | --- | --- | --- |
| Variable | Al | Ca | Fe | K | Mg | Mn | Na | S | Cu | Zn |  |
| Al | 0 | 0 | 0 | 0 | 0 | 0.148 | 0 | 0 | 0 | -0.322 |  |
| Ca | 0 | 0 | 0.586 | 0 | 0 | 0 | 0 | 0 | 0 | 0 |  |
| Fe | 0 | 0.586 | 0 | 0 | 0.514 | 0 | 0 | 0 | 0 | 0 |  |
| K | 0 | 0 | 0 | 0 | 0 | 0.741 | 0 | 0.17 | 0 | 0 |  |
| Mg | 0 | 0 | 0.514 | 0 | 0 | 0 | 0 | 0 | 0 | 0.671 |  |
| Mn | 0.148 | 0 | 0 | 0.741 | 0 | 0 | 0 | 0 | 0 | 0 |  |
| Na | 0 | 0 | 0 | 0 | 0 | 0 | 0 | 0.69 | 0 | 0 |  |
| S | 0 | 0 | 0 | 0.17 | 0 | 0 | 0.69 | 0 | 0 | 0.287 |  |
| Cu | 0 | 0 | 0 | 0 | 0 | 0 | 0 | 0 | 0 | 0.063 |  |
| Zn | -0.322 | 0 | 0 | 0 | 0.671 | 0 | 0 | 0.287 | 0.063 | 0 |  |
| **For BAVC** | | | | | | | | | | | |
| Variable | Al | Ca | Fe | K | Mg | Mn | Na | S | Cu | Zn |  |
| Al | 0 | 0 | -0.78 | 0.75 | 0.15 | 0 | 0 | 0.39 | 0 | 0.083 |  |
| Ca | 0 | 0 | 0 | 0.291 | 0 | 0 | 0 | 0 | 0 | 0 |  |
| Fe | -0.78 | 0 | 0 | 0.817 | 0 | 0 | 0 | 0 | 0 | 0.405 |  |
| K | 0.75 | 0.291 | 0.817 | 0 | 0 | 0.261 | 0 | 0 | 0 | -0.278 |  |
| Mg | 0.15 | 0 | 0 | 0 | 0 | 0 | 0 | 0 | 0 | 0.508 |  |
| Mn | 0 | 0 | 0 | 0.261 | 0 | 0 | 0.67 | 0 | 0 | -0.446 |  |
| Na | 0 | 0 | 0 | 0 | 0 | 0.67 | 0 | 0 | 0 | 0.668 |  |
| S | 0.39 | 0 | 0 | 0 | 0 | 0 | 0 | 0 | 0 | 0.312 |  |
| Cu | 0 | 0 | 0 | 0 | 0 | 0 | 0 | 0 | 0 | 0.014 |  |
| Zn | 0.083 | 0 | 0.405 | -0.278 | 0.508 | -0.446 | 0.668 | 0.312 | 0.014 | 0 |  |
| **For BESBAVC** | | | | | | | | | | | |
| Variable | Al | Ca | Fe | K | Mg | Mn | Na | S | Cu | Zn |  |
| Al | 0 | 0 | 0 | 0 | 0 | 0 | 0 | 0 | 0.036 | 0.065 |  |
| Ca | 0 | 0 | 0.661 | 0 | 0 | 0 | 0 | 0 | 0 | 0 |  |
| Fe | 0 | 0.661 | 0 | 0.329 | 0 | 0 | 0 | 0 | 0 | 0.249 |  |
| K | 0 | 0 | 0.329 | 0 | 0 | 0 | 0.528 | 0 | 0 | 0 |  |
| Mg | 0 | 0 | 0 | 0 | 0 | 0 | 0 | 0 | 0 | 0.534 |  |
| Mn | 0 | 0 | 0 | 0 | 0 | 0 | 0 | 0 | 0 | 0.476 |  |
| Na | 0 | 0 | 0 | 0.528 | 0 | 0 | 0 | 0 | 0 | 0.28 |  |
| S | 0 | 0 | 0 | 0 | 0 | 0 | 0 | 0 | 0 | 0.228 |  |
| Cu | 0.036 | 0 | 0 | 0 | 0 | 0 | 0 | 0 | 0 | 0.029 |  |
| Zn | 0.065 | 0 | 0.249 | 0 | 0.534 | 0.476 | 0.28 | 0.228 | 0.029 | 0 |  |
